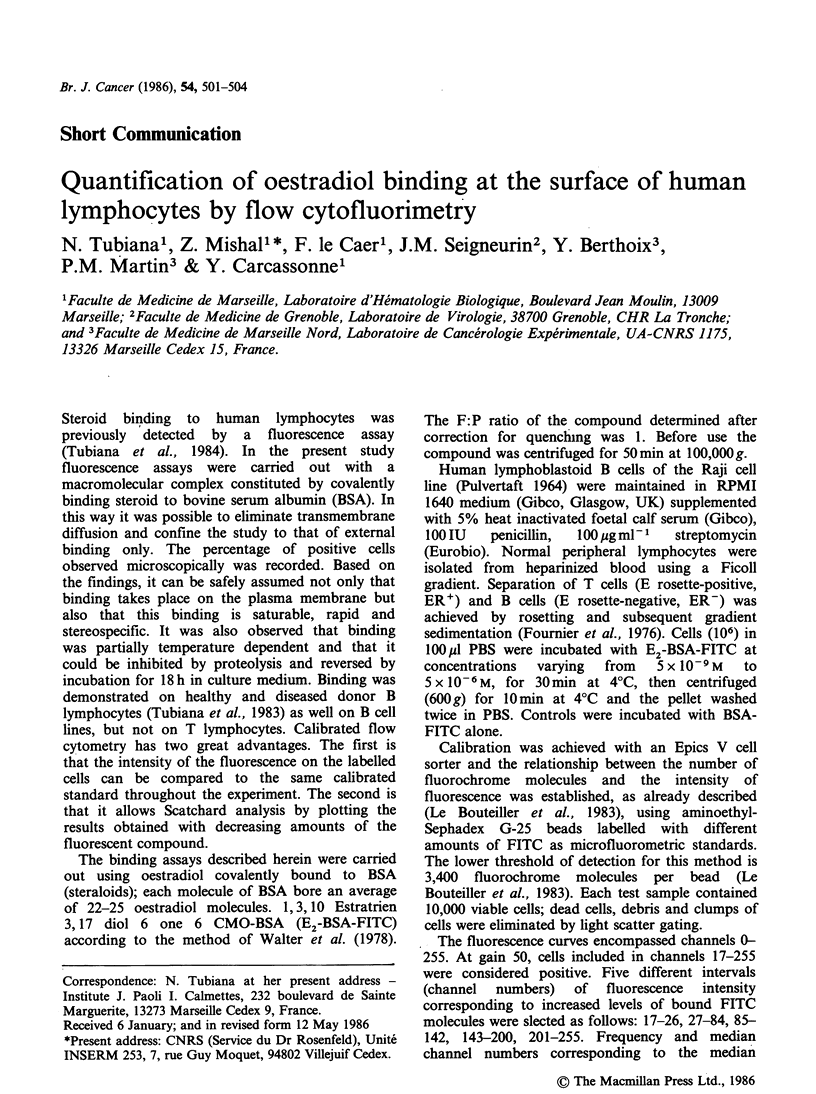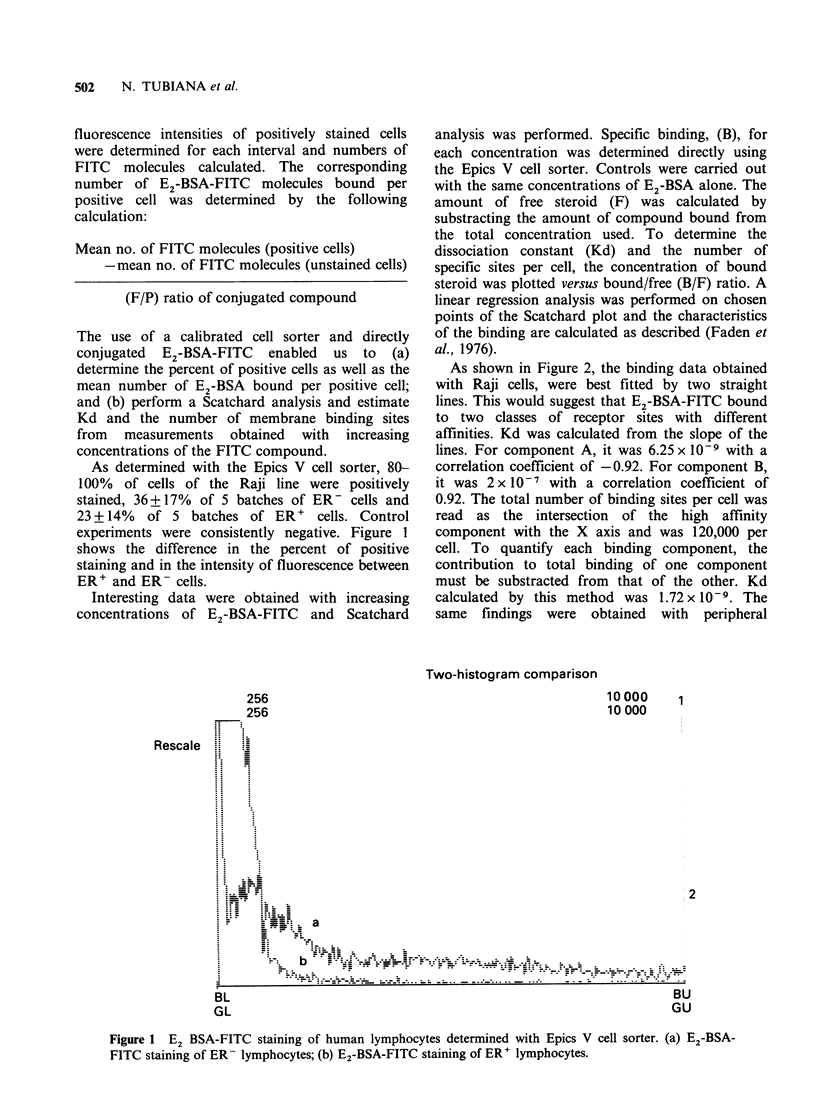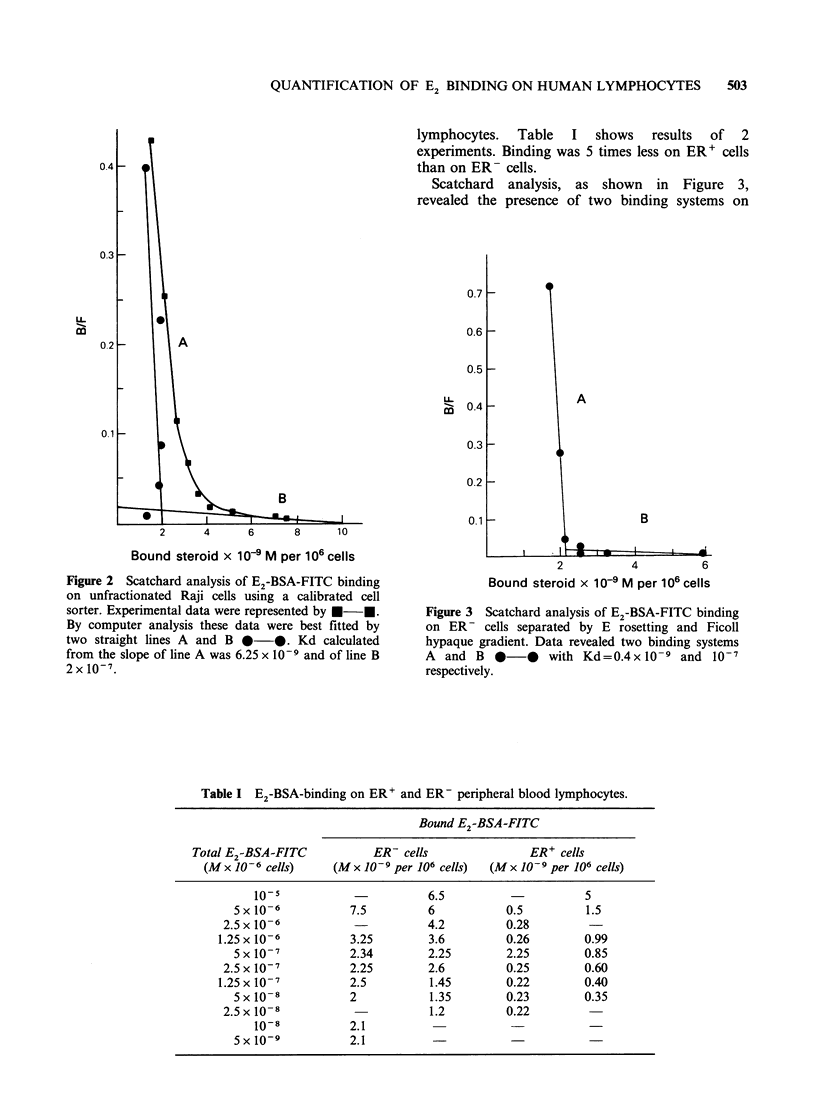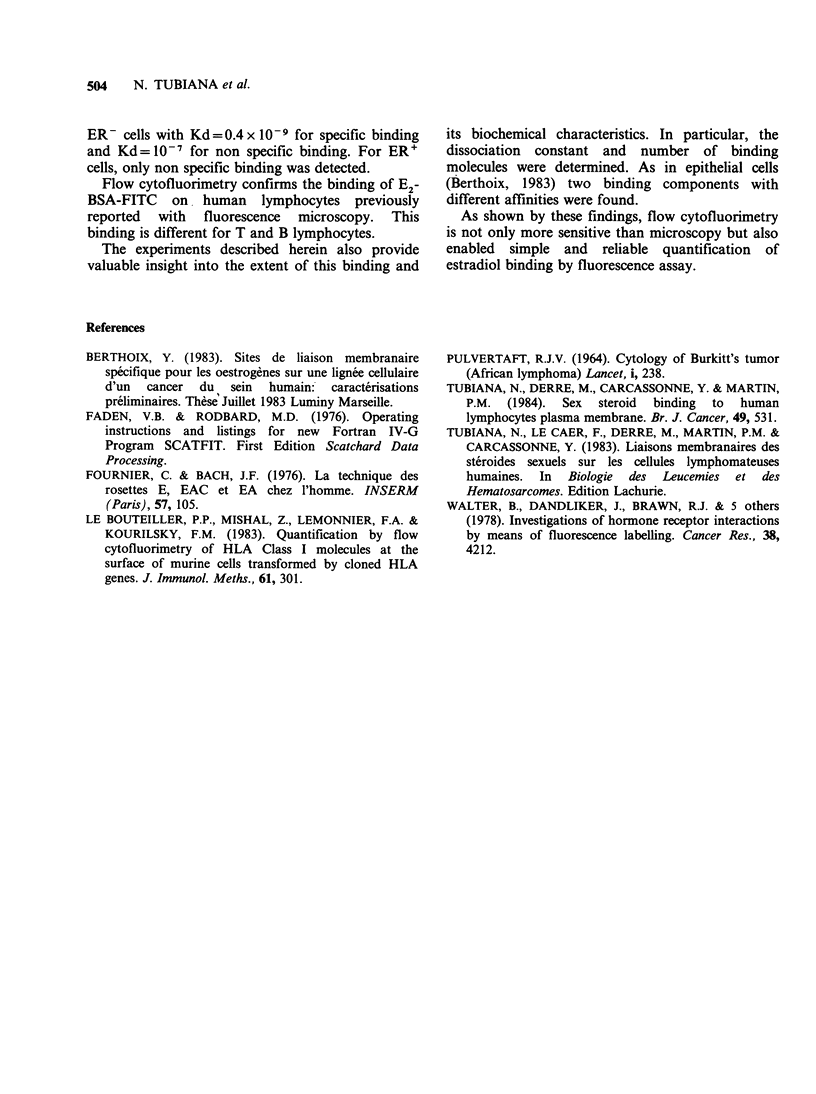# Quantification of oestradiol binding at the surface of human lymphocytes by flow cytofluorimetry.

**DOI:** 10.1038/bjc.1986.203

**Published:** 1986-09

**Authors:** N. Tubiana, Z. Mishal, F. le Caer, J. M. Seigneurin, Y. Berthoix, P. M. Martin, Y. Carcassonne


*Br. J. Cancer* (1986), **54**, 501–504

### Short Communication

### Quantification of oestradiol binding at the surface of human lymphocytes by flow cytofluorimetry

N. Tubiana[1], Z. Mishal[1]*, F. le Caer[1], J.M. Seigneurin[2], Y. Berthoix[3], P.M. Martin[3] & Y. Carcassonne[1]

[1]*Faculte de Medicine de Marseille, Laboratoire d'Hématologie Biologique, Boulevard Jean Moulin, 13009 Marseille;* [2]*Faculte de Medicine de Grenoble, Laboratoire de Virologie, 38700 Grenoble, CHR La Tronche; and* [3]*Faculte de Medicine de Marseille Nord, Laboratoire de Cancérologie Expérimentale, UA-CNRS 1175, 13326 Marseille Cedex 15, France.*

Steroid binding to human lymphocytes was previously detected by a fluorescence assay (Tubiana *et al.*, 1984). In the present study fluorescence assays were carried out with a macromolecular complex constituted by covalently binding steroid to bovine serum albumin (BSA). In this way it was possible to eliminate transmembrane diffusion and confine the study to that of external binding only. The percentage of positive cells observed microscopically was recorded. Based on the findings, it can be safely assumed not only that binding takes place on the plasma membrane but also that this binding is saturable, rapid and stereospecific. It was also observed that binding was partially temperature dependent and that it could be inhibited by proteolysis and reversed by incubation for 18 h in culture medium. Binding was demonstrated on healthy and diseased donor B lymphocytes (Tubiana *et al.*, 1983) as well on B cell lines, but not on T lymphocytes. Calibrated flow cytometry has two great advantages. The first is that the intensity of the fluorescence on the labelled cells can be compared to the same calibrated standard throughout the experiment. The second is that it allows Scatchard analysis by plotting the results obtained with decreasing amounts of the fluorescent compound.

The binding assays described herein were carried out using oestradiol covalently bound to BSA (steraloids); each molecule of BSA bore an average of 22–25 oestradiol molecules. 1, 3, 10 Estratrien 3, 17 diol 6 one 6 CMO-BSA ($E_2$-BSA-FITC) according to the method of Walter *et al.* (1978). The F:P ratio of the compound determined after correction for quenching was 1. Before use the compound was centrifuged for 50 min at 100,000 *g*.

Human lymphoblastoid B cells of the Raji cell line (Pulvertaft 1964) were maintained in RPMI 1640 medium (Gibco, Glasgow, UK) supplemented with 5% heat inactivated foetal calf serum (Gibco), 100 IU penicillin, 100 $\mu$g ml$^{-1}$ streptomycin (Eurobio). Normal peripheral lymphocytes were isolated from heparinized blood using a Ficoll gradient. Separation of T cells (E rosette-positive, $ER^+$) and B cells (E rosette-negative, $ER^-$) was achieved by rosetting and subsequent gradient sedimentation (Fournier *et al.*, 1976). Cells ($10^6$) in 100 $\mu$l PBS were incubated with $E_2$-BSA-FITC at concentrations varying from $5 \times 10^{-9}$ M to $5 \times 10^{-6}$ M, for 30 min at 4°C, then centrifuged (600 *g*) for 10 min at 4°C and the pellet washed twice in PBS. Controls were incubated with BSA-FITC alone.

Calibration was achieved with an Epics V cell sorter and the relationship between the number of fluorochrome molecules and the intensity of fluorescence was established, as already described (Le Bouteiller *et al.*, 1983), using aminoethyl-Sephadex G-25 beads labelled with different amounts of FITC as microfluorometric standards. The lower threshold of detection for this method is 3,400 fluorochrome molecules per bead (Le Bouteiller *et al.*, 1983). Each test sample contained 10,000 viable cells; dead cells, debris and clumps of cells were eliminated by light scatter gating.

The fluorescence curves encompassed channels 0–255. At gain 50, cells included in channels 17–255 were considered positive. Five different intervals (channel numbers) of fluorescence intensity corresponding to increased levels of bound FITC molecules were slected as follows: 17–26, 27–84, 85–142, 143–200, 201–255. Frequency and median channel numbers corresponding to the median

Correspondence: N. Tubiana at her present address – Institute J. Paoli I. Calmettes, 232 boulevard de Sainte Marguerite, 13273 Marseille Cedex 9, France.
Received 6 January; and in revised form 12 May 1986
*Present address: CNRS (Service du Dr Rosenfeld), Unité INSERM 253, 7, rue Guy Moquet, 94802 Villejuif Cedex.

© The Macmillan Press Ltd., 1986

fluorescence intensities of positively stained cells were determined for each interval and numbers of FITC molecules calculated. The corresponding number of $E_2$-BSA-FITC molecules bound per positive cell was determined by the following calculation:

$$\frac{\text{Mean no. of FITC molecules (positive cells)} - \text{mean no. of FITC molecules (unstained cells)}}{\text{(F/P) ratio of conjugated compound}}$$

The use of a calibrated cell sorter and directly conjugated $E_2$-BSA-FITC enabled us to (a) determine the percent of positive cells as well as the mean number of $E_2$-BSA bound per positive cell; and (b) perform a Scatchard analysis and estimate Kd and the number of membrane binding sites from measurements obtained with increasing concentrations of the FITC compound.

As determined with the Epics V cell sorter, 80–100% of cells of the Raji line were positively stained, $36 \pm 17\%$ of 5 batches of $ER^-$ cells and $23 \pm 14\%$ of 5 batches of $ER^+$ cells. Control experiments were consistently negative. Figure 1 shows the difference in the percent of positive staining and in the intensity of fluorescence between $ER^+$ and $ER^-$ cells.

Interesting data were obtained with increasing concentrations of $E_2$-BSA-FITC and Scatchard analysis was performed. Specific binding, (B), for each concentration was determined directly using the Epics V cell sorter. Controls were carried out with the same concentrations of $E_2$-BSA alone. The amount of free steroid (F) was calculated by substracting the amount of compound bound from the total concentration used. To determine the dissociation constant (Kd) and the number of specific sites per cell, the concentration of bound steroid was plotted *versus* bound/free (B/F) ratio. A linear regression analysis was performed on chosen points of the Scatchard plot and the characteristics of the binding are calculated as described (Faden *et al.*, 1976).

As shown in Figure 2, the binding data obtained with Raji cells, were best fitted by two straight lines. This would suggest that $E_2$-BSA-FITC bound to two classes of receptor sites with different affinities. Kd was calculated from the slope of the lines. For component A, it was $6.25 \times 10^{-9}$ with a correlation coefficient of $-0.92$. For component B, it was $2 \times 10^{-7}$ with a correlation coefficient of 0.92. The total number of binding sites per cell was read as the intersection of the high affinity component with the X axis and was 120,000 per cell. To quantify each binding component, the contribution to total binding of one component must be substracted from that of the other. Kd calculated by this method was $1.72 \times 10^{-9}$. The same findings were obtained with peripheral

**Figure 1** $E_2$ BSA-FITC staining of human lymphocytes determined with Epics V cell sorter. (a) $E_2$-BSA-FITC staining of $ER^-$ lymphocytes; (b) $E_2$-BSA-FITC staining of $ER^+$ lymphocytes.

**Figure 2** Scatchard analysis of $E_2$-BSA-FITC binding on unfractionated Raji cells using a calibrated cell sorter. Experimental data were represented by ■—■. By computer analysis these data were best fitted by two straight lines A and B ●—●. Kd calculated from the slope of line A was $6.25 \times 10^{-9}$ and of line B $2 \times 10^{-7}$.

lymphocytes. Table I shows results of 2 experiments. Binding was 5 times less on $ER^+$ cells than on $ER^-$ cells.

Scatchard analysis, as shown in Figure 3, revealed the presence of two binding systems on

**Figure 3** Scatchard analysis of $E_2$-BSA-FITC binding on $ER^-$ cells separated by E rosetting and Ficoll hypaque gradient. Data revealed two binding systems A and B ●—● with $Kd = 0.4 \times 10^{-9}$ and $10^{-7}$ respectively.

**Table I** $E_2$-BSA-binding on $ER^+$ and $ER^-$ peripheral blood lymphocytes.

| | *Bound $E_2$-BSA-FITC* | | | |
|---|---|---|---|---|
| *Total $E_2$-BSA-FITC ($M \times 10^{-6}$ cells)* | *$ER^-$ cells ($M \times 10^{-9}$ per $10^6$ cells)* | | *$ER^+$ cells ($M \times 10^{-9}$ per $10^6$ cells)* | |
| $10^{-5}$ | — | 6.5 | — | 5 |
| $5 \times 10^{-6}$ | 7.5 | 6 | 0.5 | 1.5 |
| $2.5 \times 10^{-6}$ | — | 4.2 | 0.28 | — |
| $1.25 \times 10^{-6}$ | 3.25 | 3.6 | 0.26 | 0.99 |
| $5 \times 10^{-7}$ | 2.34 | 2.25 | 2.25 | 0.85 |
| $2.5 \times 10^{-7}$ | 2.25 | 2.6 | 0.25 | 0.60 |
| $1.25 \times 10^{-7}$ | 2.5 | 1.45 | 0.22 | 0.40 |
| $5 \times 10^{-8}$ | 2 | 1.35 | 0.23 | 0.35 |
| $2.5 \times 10^{-8}$ | — | 1.2 | 0.22 | — |
| $10^{-8}$ | 2.1 | — | — | — |
| $5 \times 10^{-9}$ | 2.1 | — | — | — |

$ER^-$ cells with $Kd = 0.4 \times 10^{-9}$ for specific binding and $Kd = 10^{-7}$ for non specific binding. For $ER^+$ cells, only non specific binding was detected.

Flow cytofluorimetry confirms the binding of $E_2$-BSA-FITC on human lymphocytes previously reported with fluorescence microscopy. This binding is different for T and B lymphocytes.

The experiments described herein also provide valuable insight into the extent of this binding and its biochemical characteristics. In particular, the dissociation constant and number of binding molecules were determined. As in epithelial cells (Berthoix, 1983) two binding components with different affinities were found.

As shown by these findings, flow cytofluorimetry is not only more sensitive than microscopy but also enabled simple and reliable quantification of estradiol binding by fluorescence assay.

### References

BERTHOIX, Y. (1983). Sites de liaison membranaire spécifique pour les oestrogènes sur une lignée cellulaire d'un cancer du sein humain: caractérisations préliminaires. Thèse Juillet 1983 Luminy Marseille.

FADEN, V.B. & RODBARD, M.D. (1976). Operating instructions and listings for new Fortran IV-G Program SCATFIT. First Edition *Scatchard Data Processing*.

FOURNIER, C. & BACH, J.F. (1976). La technique des rosettes E, EAC et EA chez l'homme. *INSERM (Paris)*, **57**, 105.

LE BOUTEILLER, P.P., MISHAL, Z., LEMONNIER, F.A. & KOURILSKY, F.M. (1983). Quantification by flow cytofluorimetry of HLA Class I molecules at the surface of murine cells transformed by cloned HLA genes. *J. Immunol. Meths.*, **61**, 301.

PULVERTAFT, R.J.V. (1964). Cytology of Burkitt's tumor (African lymphoma) *Lancet*, **i**, 238.

TUBIANA, N., DERRE, M., CARCASSONNE, Y. & MARTIN, P.M. (1984). Sex steroid binding to human lymphocytes plasma membrane. *Br. J. Cancer*, **49**, 531.

TUBIANA, N., LE CAER, F., DERRE, M., MARTIN, P.M. & CARCASSONNE, Y. (1983). Liaisons membranaires des stéroides sexuels sur les cellules lymphomateuses humaines. In *Biologie des Leucemies et des Hematosarcomes*. Edition Lachurie.

WALTER, B., DANDLIKER, J., BRAWN, R.J. & 5 others (1978). Investigations of hormone receptor interactions by means of fluorescence labelling. *Cancer Res.*, **38**, 4212.